# Protective Effects of Carbonated Chitosan Montmorillonite on Vomitoxin-Induced Intestinal Inflammation

**DOI:** 10.3390/polym16050715

**Published:** 2024-03-05

**Authors:** Ruifan Tang, Xianghong Ju, Xueting Niu, Xiaoxi Liu, Youquan Li, Zhichao Yu, Xingbin Ma, Yuan Gao, Yin Li, Huili Xie, Qiu Zhou, Yanhong Yong

**Affiliations:** 1 Marine Medical Research and Development Centre, Shenzhen Institute of Guangdong Ocean University, Shenzhen 518120, China; 2112104109@stu.gdou.edu.cn (R.T.); juxh77@163.com (X.J.); nxt1208@163.com (X.N.); 2112104078@stu.gdou.edu.cn (Y.G.); ly12181028@163.com (Y.L.); 2112104024@stu.gdou.edu.cn (H.X.); 2112104098@stu.gdou.edu.cn (Q.Z.); 2 College of Coastal Agricultural Sciences, Guangdong Ocean University, Zhanjiang 524088, China; liuxiaoxi_06@163.com (X.L.); youquan-li@163.com (Y.L.); yujingmary@163.com (Z.Y.); mxb1984612@126.com (X.M.)

**Keywords:** carbonated chitosan montmorillonite, vomitoxin, intestinal inflammation

## Abstract

Exposure to vomitoxin (DON) can negatively impact the intestinal health of livestock and poultry, leading to compromised nutrient absorption and utilization, resulting in slowed growth and reduced production efficiency. In this study, we synthesized carbonated chitosan montmorillonite intercalation complexes (CCM) through solution precipitation. The successful formation of intercalation complexes was confirmed by examining functional groups and surface features using infrared spectroscopy and scanning electron microscopy. To assess the impact of CCM on DON-infected mice, we established an experimental mouse model of jejunal inflammation induced by DON infection. We analyzed the effects of CCM on blood biochemical and conventional indices, jejunal inflammatory factors, pathological changes, and the expression of proteins in the MAPK pathways in DON-infected mice. Our results indicate that CCM effectively mitigates the adverse effects of DON on growth performance, jejunal injury, and the inflammatory response in mice. CCM supplementation alleviated the negative effects of DON infection on growth performance and reduced intestinal inflammation in mice. Moreover, CCM supplementation successfully inhibited the activation of the mitogen-activated protein kinase (MAPK) signaling pathway induced by DON. These findings suggest that the mitigating effect of CCM on DON-induced inflammatory injury in the murine jejunum is closely linked to the regulation of the MAPK signaling pathway.

## 1. Introduction

Deoxynivalenol, commonly known as vomitoxin, is a mycotoxin produced by various Fusarium species, including Fusarium graminearum and Fusarium yellows. This group of monoterpene compounds, characterized by similar chemical structures and biological activities, is a major contaminant in food and feed crops such as corn, wheat, and barley, with detection rates reaching up to 50% [1]. Animal exposure to DON results in symptoms such as anorexia, vomiting, diarrhea, and adverse effects on immune and reproductive functions. These effects contribute to reduced feed intake, slowed growth, immune system damage, and abnormalities due to immunosuppression and immunostimulation caused by specific doses of DON [2,3,4,5]. The economic efficiency of the industry is significantly impacted by these adverse effects. Vomitoxin, due to its stable physicochemical properties, poses challenges for detoxification through physical and chemical methods and has inherent limitations, such as reduced feed nutrition, residues from decontamination processes, and secondary contamination from DON and its reaction byproducts [6]. Therefore, to protect human health, reduce animal diseases and deaths, and minimize risks, the development of mycotoxin adsorbents with high adsorption efficiency is crucial for contemporary social and economic development.

Montmorillonite, commonly present in bentonite adsorbents, is renowned for its robust adsorption capacity [7]. Surface modification is the most effective approach for enhancing the utilization of montmorillonite [8,9]. Utilizing organic cations as modifiers can reduce the surface free energy of silicate layers and enhance their compatibility with hydrophobic polymers. This modification is typically achieved by substituting exchangeable ions (e.g., Na^+^ and Ca^2+^) with positively charged organics or biomolecules [10]. Furthermore, organic modification involving amino groups can yield organic or inorganic hybrids with unique selectivity and reactivity [11,12]. In recent years, remarkable progress has been made in terms of determining the physical and engineering properties of nanomontmorillonite composites [13]. However, due to the weak polarity and poor electrophilicity of DON, the adsorption of DON by modified montmorillonite is limited. According to related evidence and investigations, there are very few adsorbent products on the market that have a superior adsorption effect on DON, highlighting the practical significance of identifying an adsorbent with strong adsorption capabilities for DON.

Chitosan (CS), derived from the naturally occurring biopolymer chitin, is abundant in various eukaryotic organisms, including crustaceans, insects, and fungi [14,15]. Chitosan and its derivatives serve as effective antimicrobial agents, demonstrating broad-spectrum activity against bacteria, yeasts, and fungi while exhibiting low toxicity toward normal mammalian cells [16,17,18,19]. The biological activity of chitosan is significantly influenced by its molecular weight, with lower weights demonstrating superior activity [19]. By inhibiting Gram-positive bacteria, chitosan neutralizes its positive charge while negatively affecting bacteria, leading to peptidoglycan hydrolysis in cell walls and the permeation of the cell surface. Conversely, when interacting with Gram-negative bacteria, chitosan attracts the outer membrane due to its positively charged surface, thereby inhibiting the transport of the cell wall [20]. Another mechanism involves the binding of chitosan to bacterial DNA, leading to the inhibition of RNA synthesis [21].

In this study, we used chitosan as the intercalating agent and montmorillonite as the matrix. The process involved organic intercalation treatment of montmorillonite and chitosan followed by carbonization to prepare carbonized chitosan–montmorillonite composites. These composites were synthesized to evaluate both mucosal protection and adsorption activities. To elucidate the potential mechanism by which CCM mitigates DON infection-induced intestinal inflammatory injury in mice, we established a DON infection model in C57BL/6 mice.

## 2. Materials and Methods

### 2.1. Animals and Management

Four-week-old male C57BL/6 mice weighing 18–20 g were purchased from Spivey Biotechnology Co., Ltd. (Beijing, China) and divided into six groups (*n* = 6 per group). The mice were housed in an SPF-grade mouse house at the Teaching Animal Hospital of Guangdong Ocean University. The mouse cages were cleaned, and bedding was replaced every two days. The mice had ad libitum access to food and water. The environmental conditions were maintained at a temperature of 20–25 °C, humidity of 40–70%, natural ventilation, and a 12-hour light–dark cycle. After seven days of acclimatization, the mice were fasted for 24 h. The six groups of mice were as follows (as shown in Table 1): the CON group, DON group (2 mg/kg), CCM group (2 mg/kg), CCM-L group (1 mg/kg), CCM-M group (2 mg/kg), and CCM-H group (3 mg/kg). The required compounds were obtained from the respective manufacturers and dissolved in ultrapure water to prepare a standard solution (1 mg/mL), which was further diluted to the desired concentration before use. This experiment was approved by the Experimental Animal Ethics Committee of Guangdong Ocean University (2022-scuec-021).

Mice were gavaged for 14 days, during which time food consumption and body weight were systematically documented for each group. At the end of this two-week period, the mice were euthanized, and peripheral blood and intestinal tissue samples were collected for subsequent analysis.

### 2.2. In Vitro Adsorption of Vomitoxin by Intercalated Complexes

#### 2.2.1. Preparation of the Intercalation Complexes

Initially, 4.0 g of CS was gently dissolved in 196 mL of 2% glacial acetic acid solution. The pH of the solution was adjusted to 5.0 with 1 mol/L sodium hydroxide solution to preserve the structure of the MMT. Then, 2.5 g of MMT was homogeneously dispersed in 100 mL of distilled water via ultrasonication for 1.5 h. The CS solution was then carefully added dropwise to the MMT suspension at a controlled rate of 50 mL/h while performing vigorously stirring. After 24 h of reaction at 60 °C, the product was rinsed with distilled water until the wash water reached neutral pH. The product precipitate was separated by filtration, and the resulting precipitate was dried at 60 °C for 48 h. The synthesized product was removed from the drying oven, ground into a powder using a glass mortar, and sieved through a 120 mesh sieve to obtain chitosan montmorillonite (CM). Carbonization of the CM via vacuum heating at 400 °C for 4 h afforded the CCM.

#### 2.2.2. Characterization of the Intercalation Complexes

The samples of montmorillonite, chitosan, and carbonized chitosan–montmorillonite intercalation complexes were investigated via infrared spectroscopy using a Fourier infrared spectrometer (Thermo Scientific iN10, Walthman, MA, USA) in the wavelength range of 400–4000 cm^−1^ using the potassium bromide compression method. The samples were prepared via drying, gold spraying, and imaging using a scanning electron microscope (SEM) (TESCAN MIRA LMS, Brno, Czech Republic). The samples were characterized using an automatic surface area and porosity analyzer (Quantachrome Nova 4000e, Boynton Beach, FL, USA) to analyze the specific surface area, pore volume, and pore size.

#### 2.2.3. In Vitro Adsorption Experiments

In vitro adsorption and desorption studies of vomitoxin were performed at a pH of 2.0 (simulating gastric fluid) or 6.5 (simulating intestinal fluid) and a temperature of 37 °C (approximating most mammalian body temperatures). Each sample was mixed with 5 mL of vomitoxin working solution at a concentration of 2 μg/mL. The sample concentrations were increased to 1 mg/mL, 2 mg/mL, and 4 mg/mL. After 2 h of complete adsorption, 1 mL of supernatant was removed from the system using a 0.22 μm membrane filter. The concentration of vomitoxin before and after adsorption was determined via high-performance liquid chromatography. All the experiments were performed in triplicate, and the mean and standard deviation were calculated.

### 2.3. In Vivo Adsorption of Vomitoxin by Intercalated Complexes

#### 2.3.1. Sample Collection

Feeding was stopped the night before sampling. Mice were restrained on a bench, and blood was collected from the eyeballs and centrifuged at 3000 rpm for 15 min at 4 °C. Serum was collected and stored at −20 °C. Jejunal samples were collected after euthanasia. Some of these samples were fixed in 4% formaldehyde solution for sectioning, and the remaining samples were stored at −80 °C.

#### 2.3.2. Complete Blood Count

A fraction of whole blood was collected in EDTA-coated tubes and immediately analyzed using an automated blood cell analyzer (5180, Ulit, Guilin, China).

#### 2.3.3. Measurement of Serum Biochemical Indicators

After thawing and centrifugation (3000× *g*, 4 °C, 15 min), 200 μL of serum was added to a biochemical analysis system (Tianjin Mnc Technologies, Tianjin, China). Serum glucose, liver function (alanine aminotransferase and glutamic oxalate aminotransferase), and total protein levels were determined and analyzed according to the manufacturer’s instructions (Tianjin Mnc Technologies, Tianjin, China).

#### 2.3.4. Enzyme-Linked Immunosorbent Assay (ELISA)

Saline was added to the intestinal samples (0.2 g), which were subsequently homogenized using a frozen tissue grinder. After centrifugation at 3000 rpm for 10 min, the supernatant was collected. The concentrations of IL-6, IL-10, IL-1β, and TNF-α in the serum and intestinal tissue supernatant were determined using a commercially available mouse ELISA kit (catalog numbers MM-0163M1, MM-0176M1, and MM-0040M1; Shanghai Enzyme Immunobio, Shanghai, China).

#### 2.3.5. Histological Examination

Jejunal intestinal segments were initially fixed in 4% formaldehyde solution for 24 h and subjected to treatment consisting of water rinsing, gradient ethanol dehydration, xylene transparency, and subsequent paraffin embedding. The samples were then sectioned at 3–4 μm and stained with hematoxylin and eosin. Areas of intestinal tissue with intact villi were identified and imaged for each section. Using Image-Pro Plus 5.0 image analysis software, the villus height and crypt depth were measured for each individual villus, and the ratio of villus height to crypt depth was calculated. Finally, these data were systematically organized and statistically analyzed.

#### 2.3.6. Western Blotting Analysis

Total protein was extracted using RIPA lysis buffer (Cowin Bio, Beijing, China), and the concentration was determined using a BCA protein assay kit (Beyotime, Shanghai, China). Equal amounts of proteolytic products were separated via sodium dodecyl sulfate–polyacrylamide gel electrophoresis and subsequently transferred to a nitrocellulose membrane (Merck Millipore, Heidelberg, Germany). After blocking with buffer (Beyotime, Shanghai, China) for 1 h, the membrane was incubated overnight at 4 °C with the following various antibodies: JNK (1:1000; Cat. No. 66210-1-AP), phospho-JNK (1:1000; Cat. No. 80024-1-AP), ERK1/2 (1:1000; Cat. No. 11257-1-AP), phospho-ERK1/2 (1:1000; Cat. No. 28733-1-AP), P38 (1:1000; Cat. No. 14064-1-AP), phospho-P38 (1:1000; Cat. No. 28796-1-AP; all from Proteintech, Wuhan, China), and β-actin (1:1000; Cat. No. 4970; Cell Signaling Technology, Danvers, MA, USA). Horseradish peroxidase-conjugated secondary antibodies (anti-rabbit IgG and anti-mouse IgG; Beyotime, Shanghai, China) were used. Positive bands were detected via enhanced chemiluminescence (ECL; Tanon, Shanghai, China). The band intensity was analyzed semiquantitatively using Gel-Pro Analyzer v. 4.0 (Meyer Instruments, Houston, TX, USA), and the relative protein expression levels were normalized to match that of β-actin.

#### 2.3.7. Statistical Analyses

All the data were derived from at least three independent experiments, and statistical significance was determined via one-way ANOVA and *t* tests. *p* < 0.05 was considered to indicate statistical significance. IBM SPSS Statistics 26 was used for all the statistical analyses. FTIR data were processed for graphing using Origin 2023, while GraphPad Prism 9 was used to generate other bar and line graphs.

## 3. Results

### 3.1. Characterization of CCM

**Figure 1:**
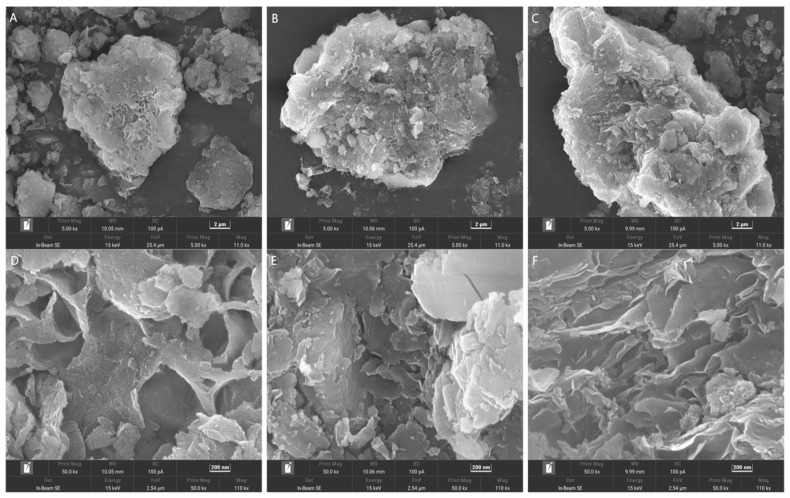
Scanning electron micrographs of (**A**,**D**) MMT, (**B**,**E**) CM, and (**C**,**F**) CCM.

Scanning electron microscopy (SEM) revealed the surface morphology of the MMT, as shown in Figure 1A. The pristine MMT exhibited a distinct lamellar structure characterized by prominent voids and layer formations conducive to the incorporation of CS. As shown in Figure 1B, CS was fragmented into smaller molecules during the reaction and integrated into the voids and layers of the MMT structure. Figure 1C and Table 2 show a remarkable increase in the pore volume and average pore size of MMT after carbonization, and the specific surface area decreased. The negatively charged surface of MMT can attract positively charged CS, promoting electrostatic interactions between the MMT and CS particles. This interaction facilitates the uniform distribution of CS particles on the MMTB surface.

**Figure 2:**
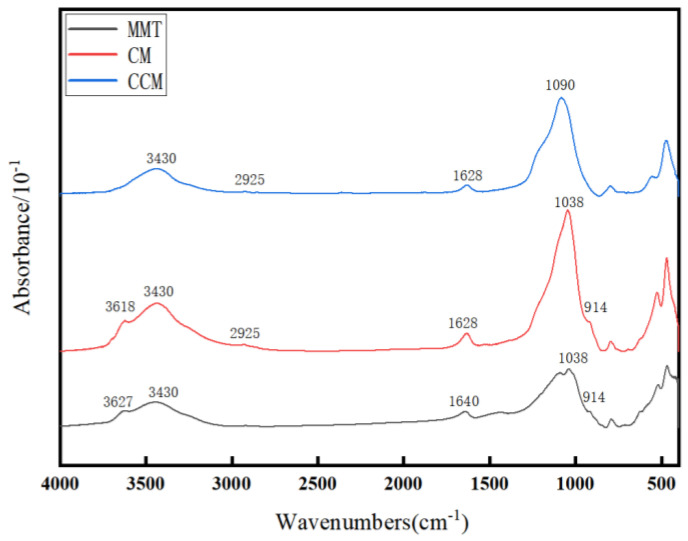
Infrared spectral detection of MMT, CM, and CCM.

FTIR spectroscopy was used to analyze the surface functional groups of MMT, CM, and CCM, as presented in Figure 2. The FTIR spectra of MMT featured peaks at 1038 cm^−1^ and 914 cm^−1^, indicative of Si-O-Si asymmetric stretching and Al-O stretching vibrations, respectively. Additionally, the bands at 3627 cm^−1^, 3430 cm^−1^, and 1640 cm^−1^ correspond to the OH stretching vibrations of the structural hydroxyl groups, physically adsorbed water, and water bending vibrations, respectively. The absorption peak at 1628 cm^−1^ is attributed to the overlap of C=O vibrations (amide I band, 1620 cm^−1^) and C-N bending vibrations (1651 cm^−1^). The prominent peak at 2925 cm^−1^, indicative of CH_2_ asymmetric stretching vibrations, suggested a high organic carbon content in CM and CCM. Therefore, the FTIR spectra confirmed the presence of numerous hydroxyl, nitrogen, and carbon-based functional groups in CM and CCM. The observation of characteristic bands for both MMT and CS indicated the successful preparation of the CS-MMT composites. Moreover, the alteration of and shift in the structural hydroxyl peaks suggest that water loss occurred during the carbonization process in CCM.

### 3.2. In Vitro Adsorption Results of DON by CCM

**Figure 3:**
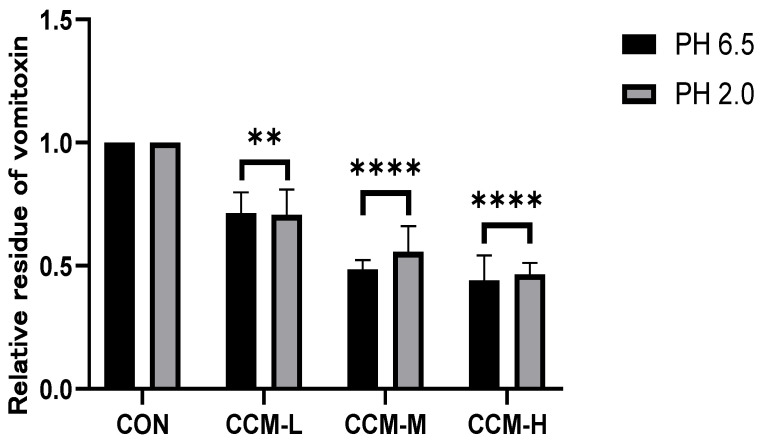
Adsorption effect of different doses of CCCM on vomitoxin at pHs 2.0 and 6.5. Compared to those in the DON group, ** *p* < 0.01 and **** *p* < 0.0001.

The adsorption of DON by the three adsorbents at varying pH levels is depicted in Figure 3. These findings indicate that in PBS solution, the adsorptions of DON by CCM at pHs 2.0 and 6.5 were dose-dependent. Approximately 55.98% of DON could be adsorbed with the addition of DON, reaching up to 4 mg/mL.

Desorption experiments were conducted with the three CCM additions to assess the stability of the adsorbent–DON complexes at pH 8.0. The results, presented in Table 3, demonstrated that the CCM-DON complexes maintained structural stability at pH 8.0, with negligible or no desorption observed.

### 3.3. Effect of CCM on the Growth Performance of Mice

**Figure 4:**
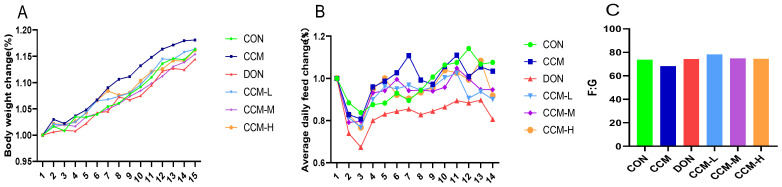
Effects of CCM on mean daily weight gain (**A**), mean daily feed intake (**B**), and the feed-to-weight ratio (**C**) in a DON-contaminated mouse model.

Figure 4 illustrates the results of an experiment assessing the impact of the oral administration (gavage) of CCM on the growth performance of mice. The data indicate that compared with CON gavage, CCM gavage increased average daily weight gain but had no significant effect on daily food intake. In contrast, the gavage of DON markedly decreased average daily feed intake by 8.07% and weight gain by 6.67% relative to those of the CON group without significantly affecting the feed-to-weight ratio. However, when DON was administered simultaneously with varying doses of CCM, there was a notable improvement in the growth performance of the mice, with significant increases in both average daily feed intake and weight gain compared to those in the DON-only group.

### 3.4. Effect of CCM on Routine Blood Indices in Mice

**Figure 5:**
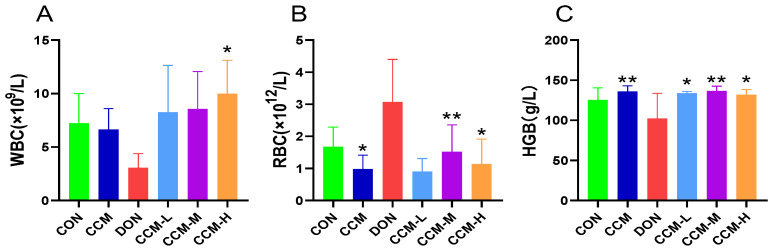
Effect of CCM on hematological parameters in DON-contaminated mice: (**A**) WBC; (**B**) RBC; (**C**) HGB. All the data are expressed as the mean ± SEM. Compared to those in the DON group, * *p* < 0.05 and ** *p* < 0.01.

Figure 5 shows the effects of CCM on the routine blood indices of DON-contaminated mice. The results indicate that oral administration of CCM did not significantly alter these blood indices compared to those of the control group. However, compared with CON treatment, DON treatment alone caused a decrease in WBC and HGB counts and an increase in the RBC count; however, these changes were not significant. Conversely, after treatment with CCM, the WBC, RBC, and HGB levels of the DON-contaminated mice significantly improved (*p* < 0.05), returning to levels comparable to those of the CON group.

### 3.5. Effect of CCM on Blood Biochemical Indices in Mice

**Figure 6:**
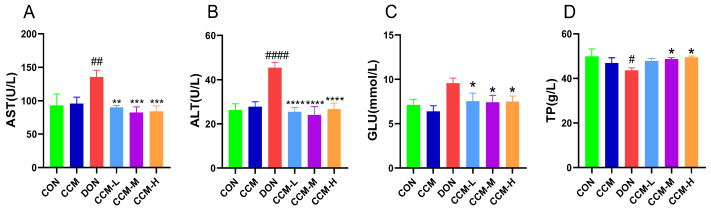
Effect of CCM on the serum biochemical indices of vomitoxin-contaminated mice: (**A**) the level of AST in mouse blood; (**B**) the level of ALT in mouse blood; (**C**) the level of GLU in mouse blood; (**D**) the level of TP in mouse blood. All the data are expressed as the mean ± SEM. Compared to those in the DON group, **** *p* < 0.0001, *** *p* < 0.001, and ** *p* < 0.01. * *p* < 0.05. Compared to those of the CON group, #### *p* < 0.0001, ## *p* < 0.01, and # *p* < 0.05.

Figure 6 displays the outcomes of the study assessing the impact of CCM on serum biochemical markers in mice. According to the findings, the oral administration of CCM did not significantly affect the levels of these serum markers compared to those in the control group. However, the administration of DON led to a significant reduction in the serum TP concentration (*p* < 0.05) and significant increases in the AST, ALT, and GLU concentrations (*p* < 0.05). Post-CCM treatment, the serum AST, ALT, and GLU levels in DON-treated mice significantly improved (*p* < 0.05), returning to values similar to those of the CON group.

### 3.6. Histopathological Changes in the Jejunum Due to DON Inhibition by CCM

**Figure 7:**
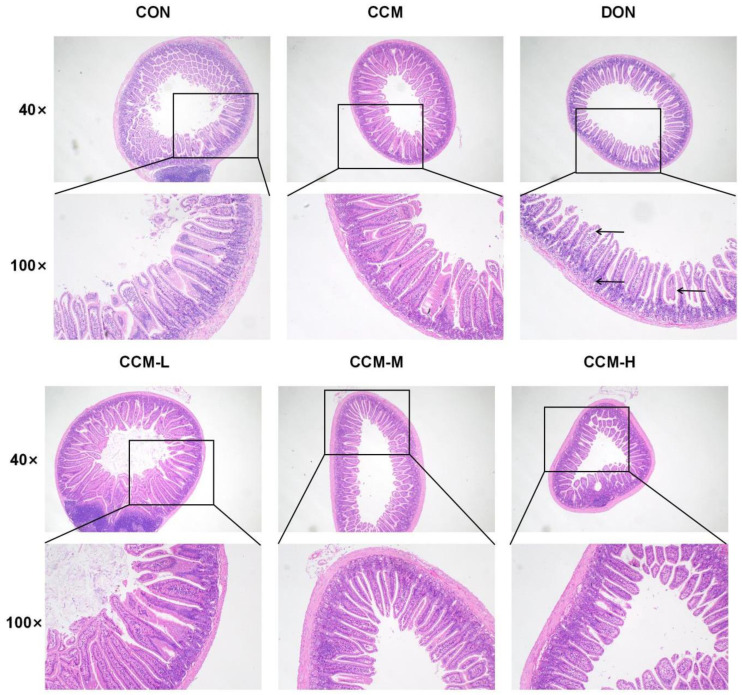
Histological changes in jejunal tissue (×40, ×100) caused by CCM in DON patients.

Figure 7 presents the experimental findings related to the impact of CCM on mouse jejunal tissues. Oral administration of CCM did not significantly affect these tissues compared to those of the control group. However, the administration of DON markedly reduced the villus height, crypt depth, and number of goblet cells in the mouse jejunum, as indicated by the observation of inflammatory cell infiltration. However, the inclusion of CCM significantly mitigated these histopathological symptoms of jejunal enteritis. In addition, the histological sections obtained following CCM treatment were not significantly different to those obtained from the CON group, and the structural damage to the jejunum was significantly improved.

### 3.7. CCM Inhibits DON-Induced Intestinal Inflammation in Mice

**Figure 8:**
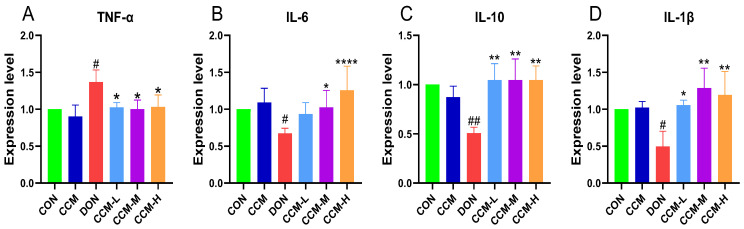
Effect of CMM on inflammatory factor expression in the mouse jejunum: (**A**) mouse jejunal TNF-α levels; (**B**) mouse jejunal IL-6 levels; (**C**) mouse jejunal IL-10 levels; (**D**) mouse jejunal IL-1β levels. All the data are expressed as the mean ± SEM. Compared to those in the DON group, **** *p* < 0.0001, ** *p* < 0.01, and * *p* < 0.05. Compared to the CON group, ## *p* < 0.01 and # *p* < 0.05.

Figure 8 shows the experimental outcomes regarding the impact of CCM on inflammatory factors in the jejuna of mice. Oral administration of CCM did not significantly influence the levels of these inflammatory markers compared to those in the control group. However, the administration of DON resulted in a significant increase in the level of TNF-α in the mouse jejunum (*p* < 0.05) and a significant reduction in the levels of IL-1β and IL-6 (*p* < 0.05). The level of IL-10 strongly decreased (*p* < 0.01). The detrimental effects of DON were significantly mitigated when CCM was administered simultaneously, and all inflammatory markers were restored to normal levels; moreover, there was no significant difference in the CCM-treated group compared to the CON group (*p* > 0.05).

### 3.8. CCM Inhibits Activation of the MAPK Signaling Pathway

**Figure 9:**
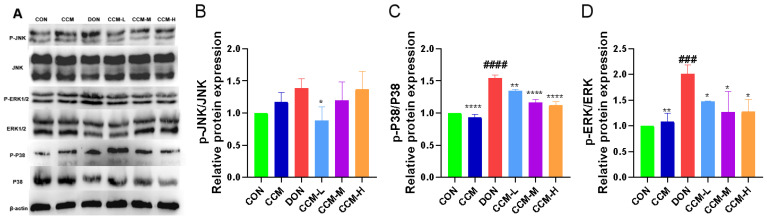
Effect of CCM on the expression of proteins related to the MAPK signaling pathway in mice. The protein expression of p-P38, P38, p-ERK, ERK, p-JNK, and JNK was quantified via protein blotting (**A**) and phosphorylation (**B**–**D**). All the data are expressed as the mean ± SEM. Compared to those in the DON group, **** *p* < 0.0001, ** *p* < 0.01, and * *p* < 0.05. Compared to the CON group, #### *p* < 0.0001 and ### *p* < 0.001.

Figure 9 shows the results concerning the impact of CCM on the expression of proteins in the MAPK pathway in the jejunal tissue of mice. The findings revealed that oral administration of CCM did not significantly alter the expression of proteins in this pathway compared to that in the control group. However, compared with those in the CON group, the phosphorylation of the P38 and ERK proteins in the DON-treated mice was notably greater (*p* < 0.001). Similarly, the expression of the phosphorylated JNK protein was also increased but not to a significant extent. Moreover, after CCM treatment, there was a significant reduction in the phosphorylation of these MAPK pathway proteins in DON-infected mice (*p* < 0.05), consistent with the results observed in the CON group. However, there was no significant change in the degree of JNK protein phosphorylation in the CCM-M or CCM-H groups (*p* > 0.05).

## 4. Discussion

Silicoaluminate minerals are predominantly utilized as mycotoxin adsorbents. This class of substances chiefly comprises layered silicoaluminates, such as bentonite, montmorillonite, kaolin, illite, and zeolites. The adsorption principle hinges on their large specific surface area, a hydrophilic negatively charged surface, and the presence of numerous natural nanomicropores within their structure that facilitate homogenous substitution. This structure, which contains abundant exchangeable cations between layers, is well suited for adsorbing mycotoxins that possess polar groups, such as aflatoxins [22]. However, due to the weak polarity and poor electrophilicity of DON, silicoaluminate adsorbents struggle to bind to DON via charge adsorption [23]. Studies have shown that the adsorption of DON by natural silicoaluminate adsorbents is pH-dependent, allowing only limited DON adsorption at pH 3.0 and reaching a maximum adsorption efficiency of only 50% depending on the mineral [24]. Researchers have attempted to enhance the adsorption capacity of silica aluminate for low-polarity mycotoxins by employing organic modification, acid activation, and complex loading; however, the results have been inconclusive [25]. Yanbing Wu reported that the equilibrium adsorption amount and efficiency of modified nonmetallic mineral adsorbents for DON were significantly lower than those before modification. Techniques such as organic anion modification of silica–alumina salts and blending of different kinds of silica–alumina salts failed to improve the low adsorption of DON [26]. In this study, a chitosan–montmorillonite intercalation complex was successfully synthesized using the solution precipitation method, which, after high-temperature carbonization, yielded a CCM. The characterization of the CCM samples using Fourier transform infrared spectroscopy and SEM confirmed the successful formation of these intercalation complexes. Adsorption test results showed that CCM was appropriate for DON adsorption in aqueous solution, achieving an adsorption rate of up to 55.98% under high-dose conditions. While the binding efficacy of CCM to DON was somewhat influenced by external conditions, its performance exceeded that of traditional montmorillonite. The complex formed by CCM and DON was structurally stable and resistant to desorption.

Feeding animals toxin-containing feed can lead to rapid toxic reactions, chronic toxic reactions, and even death. However, in actual production, mycotoxins primarily affect animals not only by causing specific disease symptoms but also by negatively impacting their metabolism and physiological functions through prolonged low-dose exposure, subsequently affecting their productivity [23]. In one study, weaned piglets fed a naturally moldy diet supplemented with DON (with a concentration of 3.78 mg/kg) exhibited a significant decrease in average daily weight gain of 14.00% (*p* < 0.05) compared to that of the control group fed a basal diet. Moreover, the feed-to-weight ratio increased significantly by 14.35% (*p* < 0.05), while there was no significant difference in the average daily feed intake [27]. According to a 2009 EU survey report on toxin adsorbents, only activated carbon displayed effective adsorption of DON, with varying degrees of adsorption depending on the type of activated carbon. Another study revealed that the addition of bamboo charcoal and bamboo vinegar solution to moldy diets did not significantly ameliorate the negative effect of DON on the average daily weight gain of weaned piglets. However, the average daily feed intake in the bamboo charcoal group was 2.25% lower than that in the moldy ration group (*p* < 0.05) and 3.66% lower than that in the control group (*p* < 0.05). The addition of bamboo charcoal and bamboo vinegar solution did not significantly improve the unfavorable effect of DON on the feed-to-weight ratio of weaned piglets [28]. In the present study, three different amounts of CCM were added to assess the impact of CCM on the growth performance of mice gavaged with DON. The results indicated that the gavage of CCM did not negatively affect the growth performance of the mice. Conversely, the gavage of DON adversely affected murine growth indices but did not significantly affect the feed-to-weight ratio (*p* > 0.05). In contrast, simultaneously gavaging CCM and DON significantly improved the average daily weight gain and average daily feed intake (*p* < 0.05) and reduced the feed-to-weight ratio in the experiment. At the end of the experiment, there were no significant differences in the average daily weight gain, average daily feed intake, or feed-to-weight ratio among the control, CCM, or treatment groups.

Extensive research has demonstrated the pronounced toxic effects of DON on animal hepatocytes. The levels of alanine transaminase (ALT) and aspartate transaminase (AST)—critical liver enzymes—are key indicators of liver function. Typically, ALT and AST levels in the blood remain stable; however, liver damage or dysfunction causes these enzymes to be released into the bloodstream, increasing the serum ALT and AST levels [29,30]. Several studies have reported that the addition of different doses of DON results in increased levels of alkaline phosphatase (ALP) and alanine transaminase (ALT), which are markers of liver injury, in mice [31]. The present study showed that the gavage of mice with a diet containing 2 mg/kg DON resulted in a significant increase in the serum GLU, AST, and ALT levels (*p* < 0.05) and a notable decrease in total protein (TP) activity (*p* < 0.05). After treatment with CCM, all the altered blood indices returned to normal, suggesting that CCM effectively mitigated DON-induced liver damage and impaired protein synthesis in mice. Additionally, DON-induced stress in mice was reflected by elevated serum glucose levels. However, intervention with CCM significantly reduced these adverse effects, normalizing the GLU, TP, AST, and ALT indices. Moreover, in our experiments, DON infection led to an increase in the number of erythrocytes and a decrease in the number of leukocytes in the blood of the mice, possibly due to the hematological disturbances caused by vomitoxin [32].

DON can act both as an immunostimulatory agent and an immunosuppressive agent, depending on various factors, such as toxin dosage, exposure duration, administration route, and the presence of other immunost stimulants [5]. Low doses of DON influence immune function by rapidly upregulating the expression of cytokine chemokines and other proinflammatory proteins while stimulating mononuclear macrophages [33,34]. Conversely, high doses of DON can cause immune system suppression by promoting leukocyte apoptosis. Previous studies have indicated that DON exposure elevates the serum levels of IL-1 and IL-6 and reduces the plasma level of TNF-α in young rabbits and broiler chickens. Other research has shown that DON exposure increases the serum levels of IL-6, TNF-α, and IFN-γ in mice but does not affect the levels of IL-1β, IL-2, or IL-4 [35]. Several studies have reported transient elevations in the serum levels of IL-10, IL-8, and TNF-α in pigs following DON injection [36]. In the present study, DON administration to mice increased TNF-α levels and decreased IL-1β, IL-6, and IL-10 levels in the mouse intestine, but these levels were restored to the control levels following treatment with CCM. We attribute these disparities to the responses of the diverse animal populations used in various studies and the differing routes and doses of DON administered.

DON impacts cellular processes by altering its molecular structure upon cell entry, binding to ribosomes, and disrupting its spatial structure. This interference inhibits peptidyl transferase, affecting the physiological function of the ribosome and, ultimately, hindering protein synthesis [37,38]. Additionally, DON induces the phosphorylation of eukaryotic initiation factor 2α via protein kinase R activation in ribosomes, leading to ribosomal stress and the activation of intracellular mitogen-activated protein kinases (MAPKs), thereby influencing various cellular physiological activities [39,40]. DON-induced cytotoxicity in piglet hippocampal neurons occurs through apoptosis induced via the MAPK signaling pathway [41]. Similarly, inflammatory responses in IPEC-J2 cells have been reported, where DON phosphorylates components of the MAPK pathway [42]. The MAPK signaling pathway is crucial for the cellular response to external changes and regulates cell growth, differentiation, apoptosis, and other key biological processes [43]. The MAPK family includes ERK (extracellular signal-regulated kinase), JNK (c-Jun N-terminal kinase), and p38 MAPK, each of which plays distinct roles in cellular processes such as proliferation, stress response, and apoptosis. The activation of these kinases primarily depends on phosphorylation. Upon specific cellular signals, the MAPK pathway activates MAPKKK (MAPK kinase kinase), which then phosphorylates and activates MAPKK (MAPK kinase), leading to the activation of ERK, JNK, or p38, amplifying and transmitting the initial signal internally and triggering corresponding biological effects [44,45]. Thus, the phosphorylation of ERK, JNK, and p38 is a key step in MAPK pathway activation and a significant marker of signaling. In our experiments, the gavage of DON in mice increased the phosphorylation of the p38, ERK, and JNK proteins, while CCM treatment significantly reduced the phosphorylation of these MAPK pathway proteins in the jejuna of DON-treated mice, restoring them to normal levels. These findings suggested that CCM treatment can inhibit DON-induced activation of the MAPK signaling pathway and may be the mechanism via which CCM alleviates DON-induced inflammatory responses.

However, several aspects still require further exploration in our study, including the interactions of adsorbents with other types of nutrients in the feed and their broader effects on livestock organisms. Additional studies are necessary to clarify these areas.

## 5. Conclusions

In our study, we confirmed the adsorption effect of CCM on DON and observed that the addition of CCM effectively alleviated the effects of DON on the growth performance and blood biochemical indices of mice, attenuated damage to the jejunum, and inhibited the phosphorylation of MAPK signaling pathway proteins. Our study further corroborated the alleviating effects of CCM on DON-induced intestinal inflammation in mice. The underlying mechanisms of the mitigating effect of CCM have also been revealed, providing fresh insights to enable comprehensive research into the development of toxin adsorbents for DON.

## Figures and Tables

**Table 1:** Grouping and treatment of mice.

Group	DON Treatment	CCM Processing
CON	-	-
DON	2 mg/kg	-
CCM	-	2 mg/kg
CCM-L	2 mg/kg	1 mg/kg
CCM-M	2 mg/kg	2 mg/kg
CCM-H	2 mg/kg	4 mg/kg

**Table 2:** The specific surface area and porosity of CCM.

Group	Specific Surface Area	Pore Volume	Average Pore Size
CM	21.745 m^2^/g	3.794 nm	10.78 nm
CCM	18.786 m^2^/g	3.969 nm	15.18 nm

**Table 3:** Desorption of vomitoxin by CCM at pH 8.0.

Group	CCM-L	CCM-M	CCM-H
Desorption Ratio (%)	-	-	0.8

## Data Availability

The data presented in this study are available upon request from the corresponding author.
